# Bucking the Trend in Wolf-Dog Hybridization: First Evidence from Europe of Hybridization between Female Dogs and Male Wolves

**DOI:** 10.1371/journal.pone.0046465

**Published:** 2012-10-03

**Authors:** Maris Hindrikson, Peep Männil, Janis Ozolins, Andrzej Krzywinski, Urmas Saarma

**Affiliations:** 1 Department of Zoology, Institute of Ecology and Earth Sciences, University of Tartu, Tartu, Estonia; 2 Estonian Environment Information Centre, Tartu, Estonia; 3 State Forest Research Institute “Silava”, Salaspils, Latvia; 4 Wildlife Park Kadzidlowo, Ruciane Nida, Poland; Barnard College, Columbia University, United States of America

## Abstract

Studies on hybridization have proved critical for understanding key evolutionary processes such as speciation and adaptation. However, from the perspective of conservation, hybridization poses a concern, as it can threaten the integrity and fitness of many wild species, including canids. As a result of habitat fragmentation and extensive hunting pressure, gray wolf (*Canis lupus*) populations have declined dramatically in Europe and elsewhere during recent centuries. Small and fragmented populations have persisted, but often only in the presence of large numbers of dogs, which increase the potential for hybridization and introgression to deleteriously affect wolf populations. Here, we demonstrate hybridization between wolf and dog populations in Estonia and Latvia, and the role of both genders in the hybridization process, using combined analysis of maternal, paternal and biparental genetic markers. Eight animals exhibiting unusual external characteristics for wolves - six from Estonia and two from Latvia - proved to be wolf-dog hybrids. However, one of the hybridization events was extraordinary. Previous field observations and genetic studies have indicated that mating between wolves and dogs is sexually asymmetrical, occurring predominantly between female wolves and male dogs. While this was also the case among the Estonian hybrids, our data revealed the existence of dog mitochondrial genomes in the Latvian hybrids and, together with Y chromosome and autosomal microsatellite data, thus provided the first evidence from Europe of mating between male wolves and female dogs. We discuss patterns of sexual asymmetry in wolf-dog hybridization.

## Introduction

The steady growth of human populations worldwide has resulted in the expansion of human-occupied territory, a decrease in suitable habitats for wildlife, and closer proximity between humans and wild animals. These trends, coupled with the increasing number of domesticated animals accompanying humans, mean that the potential for hybridization between wild and closely related domestic animals is increasing, which is especially relevant for domesticated dogs and wild canids.

There is growing evidence that many animal species can hybridize: current estimates indicate that at least 6% of European mammal species undergo some degree of hybridization [1]. Usually, the incidence of hybridization is believed to be low and its population level impact minor; however, where introgression occurs, a significant number of maladapted genetic variants can enter parental populations and may even drive species to extinction [2]. Hybridization yielding viable offspring can occur between all species in the genus *Canis* [3], indicating incomplete reproductive isolation. Moreover, hybridization coupled with subsequent introgression is a documented threat to a number of canids, including the Ethiopian wolf (*Canis simensis*) [4], the red wolf (*C. rufus*) [5], [6] and the dingo (*C. lupus dingo*) [7]. As a result of extensive hunting pressure and habitat loss during recent centuries, certain gray wolf (*Canis lupus*, subsequently referred to as ‚wolf') populations in Europe and elsewhere have dramatically decreased in size and have become increasingly fragmented. Remaining populations are also exposed to increasing numbers of humans and dogs [8]. Phylogenetic studies place dogs and wolves as sister taxa (e.g. [9]), and there is abundant evidence that dogs are a domesticated form of wolf (e.g. [10], [11]). Gray wolves and domestic dogs possess identical karyotypes and can hybridize to produce fertile offspring in the wild [12], [13]. The main conservation concern related to hybridization between wolves and domestic dogs is the significant reduction or loss of specific adaptations that could lead to the extinction of already small and fragmented wolf populations if introgression is sufficiently frequent. Therefore, monitoring genetic diversity [14], identifying the degree of hybridization and the role of females and males of both species in the hybridization process, as well as assessing the impact of hybridization on parental populations are all crucial steps for wolf conservation.

To investigate hybridization, specific genetic methods to detect the degree and direction of gene flow between wolves and dogs have been developed. Animals identified as probable hybrids on the basis of morphology have been the subject of genetic investigation in several countries. In Europe, there is genetic evidence of hybridization from Bulgaria [15], Latvia [16], Italy [17], [18], Scandinavia [19] and Iberian Peninsula [20]. This evidence has most often been based on mtDNA and autosomal microsatellite variation [16]–[21], with only two studies additionally using Y chromosome data [19], [20] to investigate the role of both genders in the hybridization process.

Field observations have only reported hybridization between female wolves and male dogs [22]–[25] and to date, there is no direct evidence from Europe of hybridization in the opposite direction, *i.e.* between male wolves and female dogs. Genetic studies of wolf-dog hybrids have also supported this sexually asymmetric pattern of hybridization [15], [16], [18]–[21], [26]; the only exception being a recent study by Munoz-Fuentes *et al.* [27] in North-America, where dog mtDNA was found in historical wolf samples from the Vancouver Island population in Canada. Thus, previous studies have suggested that the mating between wolves and dogs is sexually asymmetric, occurring predominantly, but not exclusively, between female wolves and male dogs. Asymmetric hybridization appears to be common in *Canis*, though the direction of gene flow between the sexes differs depending upon the particular pair of interacting taxa [13]. Whereas female wolves seem to participate most frequently in hybridization with dogs, hybridization between wolves and coyotes most commonly involves male wolves [28]. Meanwhile, mating between dogs and Ethiopian wolves predominantly involves male dogs and female Ethiopian wolves [4].

Wolf populations in Estonia and Latvia have been under a strong hunting pressure during the last century. By the mid-1990s, the number of wolves hunted annually was 100–300 in Estonia and over 300 in Latvia [16], sometimes constituting more than half of the estimated population. Such severe hunting pressure has resulted in dynamic fluctuations in population size and range in both countries. The most recent population size low — 15–46 hunted animals in Estonia and 115–150 in Latvia — was documented during 2001–2007 (according to Estonian Ministry of Environment the number of hunted animals reflects the population trends more reliably compared to census data). Wolf-dog hybrids have been documented in Latvia, where a litter of seven potentially hybrid pups was found in Aloja, northern Latvia in 1999, and hybridization was subsequently confirmed using genetic tests [16]. However, wolf-dog hybrids have not previously been recorded in Estonia.

In this study we used combined genetic analysis of mtDNA control region (1134 bp), 11 autosomal and 7 Y chromosome microsatellite loci to investigate putative hybrids, wolves and dogs in Estonia and Latvia. With these data, we aimed to document the occurrence of wolf-dog hybrids and the role of females and males of both species in the hybridization process.

## Materials and Methods

### Samples

**Figure 1:**
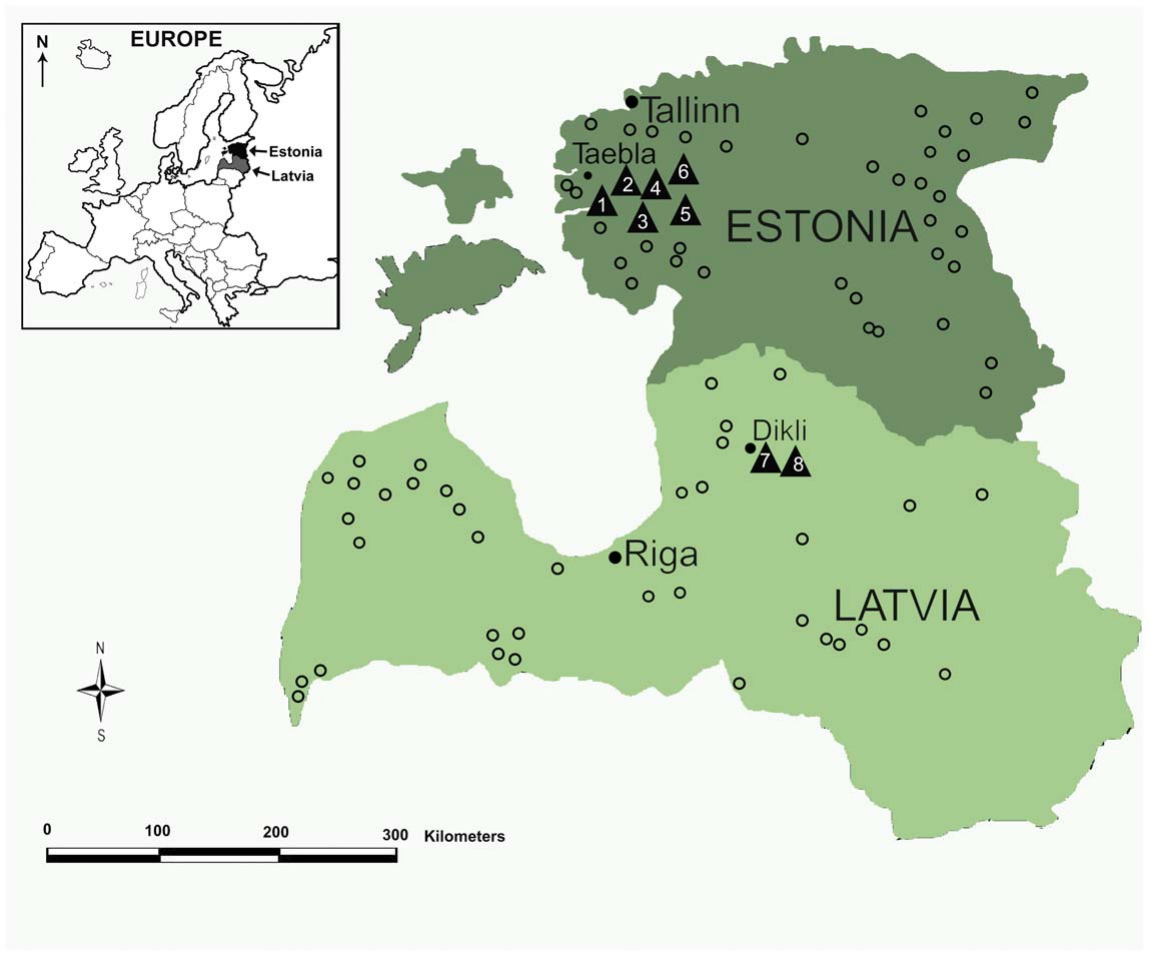
Sampling locations of wolves (small open circles) and wolf-dog hybrids (black triangles 1–8, see also **Table 1**) in Estonia (Taebla) and Latvia (Dikli), hunted during the winter period of 2008–2009. The approximate ranges of wolves are with dark green (Estonia) and light green (Latvia).

Muscle tissue samples were collected from eight putative wolf-dog hybrids: six in Estonia (Läänemaa, Taebla, winter 2008–2009) and two in Latvia (Dikli, Nov. 2008) (Fig. 1; Table 1). Muscle tissue samples of wolves (n = 74) were collected across the species range in Estonia (n = 37) and Latvia (n = 37) during 2001–2009 (Fig. 1), and blood samples from dogs (n = 21) were obtained from local veterinarians in Estonia. All samples from wild populations were collected from animals legally harvested by hunters for purposes other than this project. Blood and muscle tissue samples were stored at −20°C prior to analysis. DNA was extracted from 20–50 mg of muscle tissue or from 200 µL blood using High Pure PCR Template Preparation Kit (Roche).

**Table 1:** List of sampled wolf-dog hybrids from Estonia (Ehy) and Latvia (Lhy).

Sample	Sampling location	Gender
Ehy1	Estonia, Taebla	female hybrid
Ehy2	Estonia, Taebla	female hybrid
Ehy3	Estonia, Taebla	male hybrid
Ehy4	Estonia, Taebla	male hybrid
Ehy5	Estonia, Taebla	female hybrid
Ehy6	Estonia, Taebla	female hybrid
Lhy1	Latvia, Dikli	female hybrid
Lhy2	Latvia, Dikli	female hybrid

**Figure 2:**
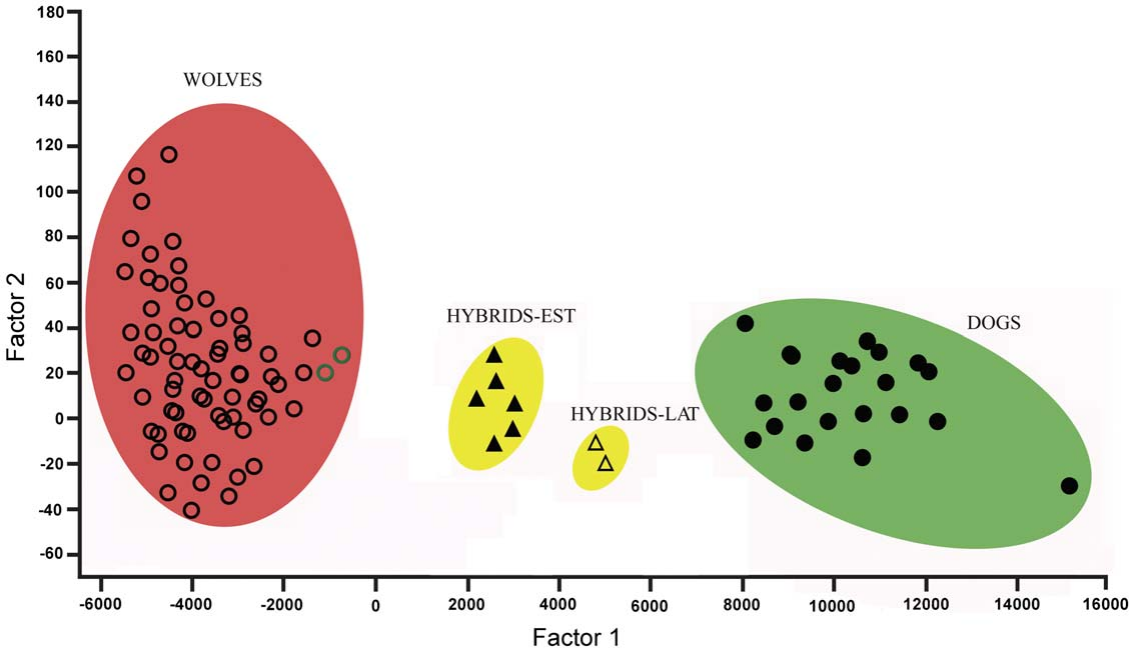
Factorial correspondence analysis of autosomal microsatellite allele data. WOLVES - Estonian and Latvian wolves; HYBRIDS-EST – wolf-dog hybrids from Estonia; HYBRIDS-LAT – wolf-dog hybrids from Latvia. Two wolves shot from the same pack with hybrids are represented by green open circles (see also Materials and Methods).

The hybrids were identified on the basis of external characteristics. The six animals from Estonia exhibited coat coloration that was atypical of wolves: four of them were unusually dark, and two were yellow. All individuals were juveniles, *i.e.* less than one year old (based on teeth and skull development) and were shot from the same large wolf pack. From this pack, two adult male wolves were also shot (these individuals clustered genetically with wolves; see Fig. 2) and one possibly subadult wolf was sighted, but escaped during the hunt. The pack territory was situated in an area of low wolf density in Western-Estonia. The two putative hybrids shot in Latvia were also juveniles from a single wolf pack and exhibited unusual yellow coats with curly fur. During the same hunt, two stray dogs were also shot nearby, though it is not clear whether they belonged to the same pack as the hybrids (these samples were not available for current study). The location of the Latvian hybrids was not far from the area used by another litter of hybrid pups in 1999 [16]. Wolf samples (animals displaying wolf-like morphology according to information from hunters) were collected at localities throughout Estonia and Latvia, including both areas where the hybrids were hunted (Fig. 1). The dog samples used included the following breeds: pedigree German Shepherd Dogs, Greyhounds, English Springer Spaniels, West Siberian Laikas and Siberian Huskies.

### Analysis of autosomal microsatellite loci

Eleven autosomal microsatellite loci were analysed: FH2001, FH2010, FH2054, FH2079, FH2088 [29], vWF [30], AHT130 [31], M-CPH2, M-CPH12 [32] and C20.253, CXX22 [33] (Table S1). The loci were chosen in order to minimise genotyping errors and null-alleles. All loci were polymerase chain reaction (PCR) amplified in a volume of 10 microlitres: 0.25 units of Amplitaq Gold (Applied Biosystems), 1 µL of 10 × concentrated PCR buffer, 2 mM MgCl_2_, 0.2 mM dNTP, 0.33 µM of primers and 10–50 ng of DNA for each reaction. PCR reaction conditions were as follows: 10 min at 94°C for initial denaturation, 11 cycles of 30 s at 94°C, 30 s at 58°C with touchdown of −0.5°C per cycle, 1 min at 72°C and 28 cycles of 30 s at 94°C, 30 s at 52°C, 1 min at 72°C and a final elongation step for 10 min at 72°C. After the PCR, the reaction mixture was diluted five times with water. To identify the length of amplified loci, 0.25 µL of the molecular size standard GeneScan™ 500 LIZ (Applied Biosystems) was added and PCR products were analysed using an ABI PRISM 3100 (Applied Biosystems) automatic sequencer following the protocol provided by the manufacturer. The alleles observed for each microsatellite were sized using Genemapper v4.0 (Applied Biosystems).

The presence of null alleles was analyzed with Micro-Checker [34]. Eighteen randomly chosen samples (15.5% of all samples) were genotyped a second time using the autosomal microsatellite loci, while eight randomly chosen male samples (17.8% of all analysed males) were genotyped a second time using the Y chromosome microsatellite loci. The results were analysed using the software Gimlet [35] to estimate the rate of different types of errors: allelic dropouts, false alleles, double errors and complete errors, as defined in Valiere [35].

We used the software Genetix 4.05.2 [36] to estimate observed (*H_O_*) [37] and unbiased expected (*H_Eunb_*) heterozygosity [38], the number of alleles (*N_A_*) and inbreeding estimator Wright's *F_IS_* [39]. Deviations from Hardy–Weinberg equilibrium were tested in wolves and dogs separately, using the program Genepop v1.2 [40]. For each population–locus combination, departure from Hardy–Weinberg expectations was assessed by exact tests with unbiased P values estimated using a Markov chain method (set to 1000 batches of 10 000 iterations each and with 10 000 steps of dememorization). A global test across loci and populations was performed using Fisher's method [41]. We also tested for linkage disequilibrium between all pairs of loci in wolves and dogs [42] using Genetix. FStat v2.9.3 was used to calculate allelic richness *A_R_* [43], using the rarefaction method [44]. Biparental multilocus genotypes were analysed using three different Bayesian approaches to estimate admixture proportions and to assign individuals to populations:

- Structure v2.2 [45] was used to evaluate the number of genetic clusters (*K*) in the data and to assign individuals to their likely origin. For identification of hybrid samples, the dataset consisted of all 103 individuals, including wolves (n = 74), dogs (n = 21) and eight hybrids from Estonia (n = 6) and Latvia (n = 2). This dataset was analysed under two different ‘Usepopinfo’ parameter settings (Table 2, A and B). Assignment of individuals into genetic clusters was performed with Structure using five MCMC runs of 5×10^5^ iterations, with the first 10% of iterations discarded as burn-in. We estimated *K* using the posterior probability of the data [Ln *P*(*D*)] as suggested by Evanno *et al.* [46]. The initial value of alpha (Dirichlet parameter for the degree of admixture) was set to 1.0 and a uniform prior for alpha was used for all populations. We used both the correlated and independent allele frequency models implemented by Falush *et al.* [47], assuming for the correlated model that for several generations following population subdivision, the evolution of allele frequencies in each population is correlated with the allele frequencies of an ancestral population and that different subpopulations have different values of *F_ST_* (prior mean of *F_ST_* for populations was set to 0.01). The value for λ (allele frequency parameter) was kept constant and fixed to 1.0.
- a Bayesian model-based clustering method for identifying hybrids was performed with Newhybrids v1.1 beta [48]. The method identifies hybrid individuals on the basis of the posterior probability of belonging to different pure parental or hybrid categories generated during n = 2 or n = 3 generations of potential interbreeding. Four distinct genotype frequency classes were simulated using Hybridlab v1.0 [49] on the basis of pure species I (Wolf) and pure species II (Dog): F1 wolf-dog hybrids (n = 100) and F2 hybrids (F1 hybrid×F1 hybrid; n = 100), including backcrosses with pure species (F1 hybrid×wolf; n = 100) and (F1 hybrid×dog; n = 100). Simulations were run with 5×10^4^ sweeps for the burn-in period and 5×10^5^ MCMC iterations. Jeffreys-like and Uniform priors were assumed for h (allele frequencies) and p (mixing proportions) in order to verify the congruence of the results.

**Table 2:** Filial generation status of wolf-dog hybrids from Estonia and Latvia, estimated with Structure v2.2 under different combinations of ‘Usepopinfo’ parameter settings.

	Parameter set A (‘Usepopinfo’ = 0 for all individuals)	Parameter set B (‘Usepopinfo’ = 1 for wolves and dogs, 0 for Estonian-Latvian hybrids	Filial generation status for hybrids
Sample			
	Membership coefficient (q) for hybrids to belong to wolf cluster		
EHy1	0.77 (0.46–1.00)	0.56 (0.38–0.73)	F1or F2?
EHy2	0.61 (0.32–0.89)	0.51 (0.34–0.69)	F1or F2?
EHy3	0.70 (0.40–0.99)	0.54 (0.36–0.72)	F1or F2?
EHy4	0.67 (0.38–0.95)	0.54 (0.36–0.71)	F1or F2?
EHy5	0.74 (0.47–1.00)	0.57 (0.39–0.74)	F1or F2?
EHy6	0.62 (0.33–0.89)	0.51 (0.33–0.69)	F1or F2?
LHy1	0.53 (0.26–0.79)	0.49 (0.38–0.73)	F1
LHy2	0.47 (0.22–0.74)	0.48 (0.31–0.65)	F1

Parameter set – two different ‘Usepopoinfo’ parameter sets (A, B) applied to the same data (n = 103). 0 – ‘Usepopinfo’ set to 0; 1 - ‘Usepopinfo’ set to 1; Ehy – hybrids from Estonia (n = 6); Lhy – hybrids from Latvia (n = 2). The q values (membership coefficient) indicate the probability of individual genotypes to belong to wolf cluster (in parentehesis 90% credible regions).

Sibling relationships and relatedness among hybrids was investigated using Kingroup v2.0 [50] and the relatedness estimator according to Konovalov and Heg [51].

Factorial correspondence analysis (FCA) implemented in Genetix [36] was used to distinguish wolves, dogs and wolf-dog hybrids on the basis of microsatellite data.

### Mitochondrial DNA analysis

Amplification of 1673 bp of mitochondrial DNA control region (mtDNA CR) was performed using newly developed primers Hu-1f (5′-TTTCATCATCATCGGACAA) and Hu-1r (5′-TTTCAGTGCCTTGCTTTA). Purified genomic DNA (20–80 ng) and 5 µM of primers were used in PCRs, which were performed in a total volume of 20 µL with 0.2 U of Advantage 2 Polymerase Mix (BD Biosciences), 2 µL of 10 × concentrated PCR buffer and 0.4 mM dNTP. Cycling parameters were: 1 min denaturing step at 95°C, followed by 10 cycles of 20 s at 95°C, 30 s at 48°C with touchdown of −0.5°C per cycle and 2 min 20 s at 68°C, then 26 cycles of 20 s at 95°C, 30 s at 43°C and 2 min 20 s at 68°C with the final elongation step of 5 min at 68°C. PCR products were purified with shrimp alkaline phosphatase/exonuclease I treatment (Fermentas). One unit of each enzyme was added to 10 µL of PCR post-reaction mix and incubated for 30 min at 37°C, followed by 15 min inactivation at 80°C. PCR products were sequenced using Big Dye Terminator cycle sequencing chemistry on an ABI PRISM 3100 (Applied Biosystems) automatic sequencer in a total volume of 10 µL, using the following steps: initial denaturation at 96°C for 1 minute, 25 cycles at 96°C for 20 s, 48°C for 15 s and final extension at 60°C for 4 minutes. Both DNA strands were sequenced with the respective primers used in DNA amplification. Consensus sequences were created using Consed [52], aligned using Clustal W [53] and checked and corrected using BioEdit [54].

A minimum spanning network was calculated with Network 4.510 using a median-joining approach [55]. The network was based on partial mtDNA control region (the final length after alignment and trimming was 1134 bp) and included wolves, dogs and hybrids from this study (from Estonia and Latvia; Table S3).

For further analysis of phylogenetic relationships between hybrids, wolves and dogs, the dataset was expanded by including additional 95 dog and 8 wolf homologous 1134 bp mitochondrial control region data from GenBank (only those that had complete 1134 bp sequence available without ambiguous sites) [56]–[59]; thus, in the final analyses 213 sequences were used (Table S3). The appropriate model of sequence evolution was calculated with jModeltest v1.0.1 using the Bayesian Information Criterion [60]. Phylogenetic trees were generated using Bayesian inference (BI) implemented in MrBayes v3.1.2 [61]. Searches were conducted with 4 simultaneous Markov Chains (3 heated and 1 cold chain) with 2 million generations, sampling every 100 generations; burn-in trees (25%) were discarded and a 50% majority rule consensus tree was calculated. To ensure that the BI was not trapped in local optima, the analysis was performed three times. Phylogenetic trees were visualized with FigTree v1.3.1 (http://tree.bio.ed.ac.uk/software/figtree).

### Analysis of Y chromosome microsatellite loci

Forty five male study animals: 25 wolves (19 from Estonia and six from Latvia), 18 dogs from diverse breeds and two wolf-dog hybrids from Estonia were also genotyped for seven Y chromosome specific microsatellite loci: MS34A, MS34B, MS41A, MS41B [62], 990-35, 650–79.2 and 650–79-3 [63] (Table S1) using the conditions described above for autosomal microsatellite loci. Based on microsatellite data from the Y chromosome loci of wolves, dogs and hybrids, a median joining network was calculated with the program Network 4.510.

## Results

### Genotyping errors

None of the analysed 103 samples included more than two loci with missing alleles. The rate of allele dropouts for autosomal microsatellite loci was 0.002, while the rate of other errors was <0.001; the respective parameters for Y chromosome loci were 0 and <0.001.

### Genetic variability

Observed heterozygosity for Estonian and Latvian wolves (n = 74) was 0.76, while allelic richness was 5.84 (Table 3). According to the Hardy-Weinberg equilibrium analysis, five loci were significantly out of equilibrium in Estonian-Latvian wolves. No indication of significant linkage disequilibrium was detected between any locus pair in any of the groups (wolves, dogs), nor across groups.

**Table 3:** Number of alleles (*N_A_*), allelic richness independent of sample size (*A_R_*), expected unbiased heterozygosity (*H_Eunb_*), observed heterozygosity (*H_O_*) and inbreeding estimator Wright's *F_IS_* for Estonian and Latvian wolves and dogs.

	Wolves (n = 74)					Dogs (n = 21)				
Locus	*N_A_*	*A_R_*	*H_O_*	*H_Eunb_*	*F_IS_*	*N_A_*	*A_R_*	*H_O_*	*H_Eunb_*	*F_IS_*
FH2010	14	4.73	0.81	0.78	−0.05	4	3.37	0.82	0.67	−0.24
FH2001	12	6.94	0.88	0.84	−0.05	7	5.17	0.59	0.74	0.19
AHT130	14	5.85	0.84	0.80	−0.06	5	4.75	0.27	0.76	0.62
FH2088	9	5.45	0.78	0.79	−0.00	8	6.34	0.32	0.68	0.52
FH2054	15	6.78	0.81	0.84	0.03	10	7.42	0.64	0.87	0.25
C20.253	9	6.37	0.82	0.81	−0.03	4	3.51	0.36	0.50	0.17
FH2079	14	4.93	0.73	0.75	0.02	4	3.29	0.23	0.54	0.57
vWF	12	5.90	0.78	0.80	0.01	3	2.00	0.14	0.13	−0.06
CPH2	6	5.19	0.62	0.78	0.20	5	4.30	0.18	0.74	0.75
CPH12	14	6.25	0.65	0.69	0.06	4	3.35	0.14	0.69	0.79
CXX22	6	4.58	0.50	0.66	0.24	2	2.38	0.46	0.51	0.09
***Mean***	*11.36*	*5.84*	*0.75*	*0.76*	*0.03*	*5.09*	*4.17*	*0.38*	*0.61*	*0.33*
***SE***	*1.00*	*0.81*	*0.03*	*0.02*	*0.03*	*0.71*	*1.65*	*0.07*	*0.06*	*0.10*

### Assignment according to variation of 11 autosomal microsatellite loci

Allele frequencies at 11 autosomal microsatellite loci recorded for all 103 individuals demonstrated that eight wolf-dog hybrids carried several alleles that were also found in wolves but were absent in dogs used in this study; for example, allele 133 at locus FH2001 and allele 222 at locus FH2010. Conversely, two alleles (167 at locus FH2054 and 105 at locus M-CPH2) were present in hybrid and dog samples but absent in Estonian and Latvian wolves (see these and other cases in Fig. S1).

In the FCA analysis, wolves, dogs and eight Estonian and Latvian hybrids could be clearly distinguished from each other based on the distributions of allele frequencies at 11 microsatellite loci (Fig. 2). The wolf-dog hybrids from Estonia and Latvia were assigned into two clusters according to their geographic location, and all eight hybrids were placed between dogs and wolves.

**Figure 3:**
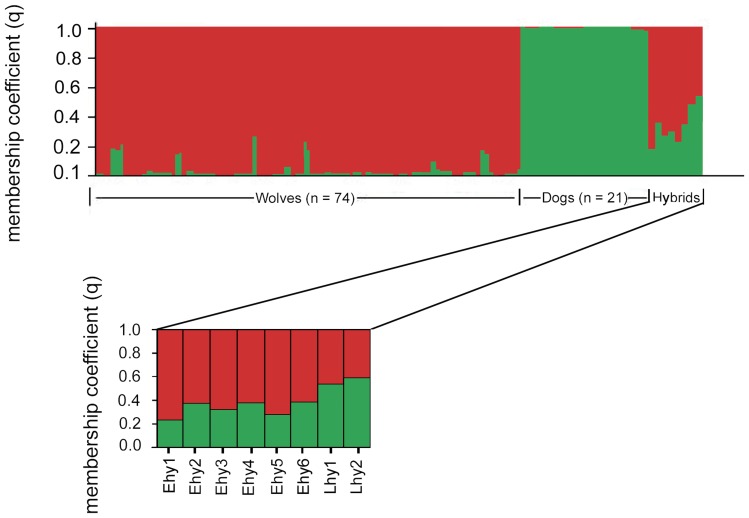
Bayesian analysis of wolf, dog and wolf-dog hybrid genotypes from Estonia and Latvia with the ‘parameter set A’ (**Table 2**). Analysis was based on 11 autosomal microsatellite loci using Structure v2.2, with the following parameters: *K* = 2 clusters; ‘Usepopinfo’ = 0 for all individuals (n = 103). Each vertical bar represents the membership coefficient (*q*) for each individual. The final eight bars on the right represent hybrids from Estonia (Ehy 1–6) and Latvia (Lhy 1–2).

**Figure 4:**
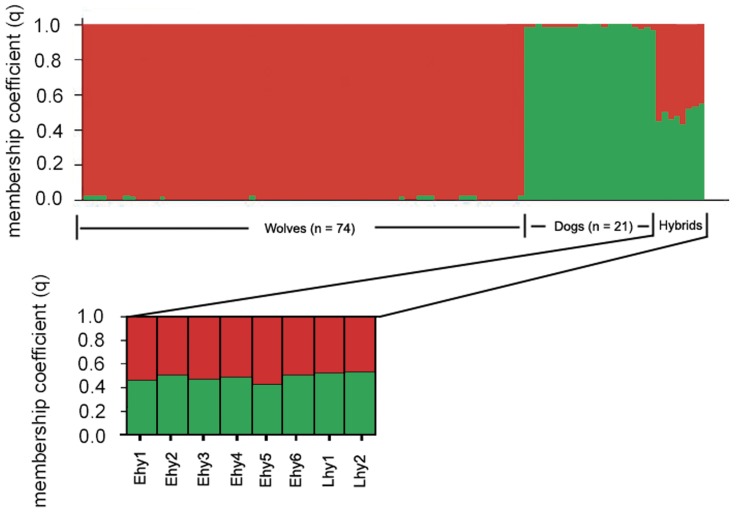
Bayesian analysis of wolf, dog and wolf-dog hybrid genotypes from Estonia and Latvia with the ‘parameter set B’ (**Table 2**). Analysis was based on 11 autosomal microsatellite loci using Structure v2.2 with the following parameters *K* = 2 clusters; ‘Usepopinfo’ = 1 for dogs and wolves, ‘Usepopinfo’ = 0 for 8 Estonian-Latvian hybrids. The final eight bars on the right represent hybrids from Estonia (Ehy 1–6) and Latvia (Lhy 1–2). Each vertical bar represents the membership coefficient (*q*) of an individual.

Assignment tests were carried out to determine whether the eight hybrids differed significantly from wolves and dogs in Estonia and Latvia. In different runs (with allele frequencies correlated or independent) used to identify putative hybrids, the number of genetic clusters estimated was always two (K = 2) according to calculations based on Evanno *et al.* [46]. With ‘parameter set A’, hybrids from Estonia were assigned with somewhat higher values to the wolf cluster, whereas hybrids from Latvia had similar probability values of belonging to wolf and dog clusters (Table 2; Fig. 3). However, with ‘parameter set B’ (Table 2), all eight hybrids from Estonia and Latvia were assigned with similar probability values to both wolf and dog clusters (Table 2, Fig. 4). Estonian-Latvian wolves exhibited membership coefficients (*q*≥0.72; 90% credible regions 0.62–1.00) of belonging to the wolf cluster.

Tests to determine whether the hybrid animals were of F1 or F2 generation hybrids with software Newhybrids assigned all Estonian and Latvian hybrids to one of three genotype frequency classes: F1 and F2 (F1×F1) hybrids or backcrosses with wolf (F1×wolf) (with probabilities to belong to assigned genotype frequency class 0.40–0.77).

According to the kinship analysis, some of the Estonian hybrids were full siblings, while others were more distantly related (Table S2). Therefore, it is possible that Estonian hybrids were not descendants of same parents. The Latvian hybrids were full siblings.

### Matrilineal phylogenies based on mtDNA control region haplotypes (1134 bp)

**Figure 5:**
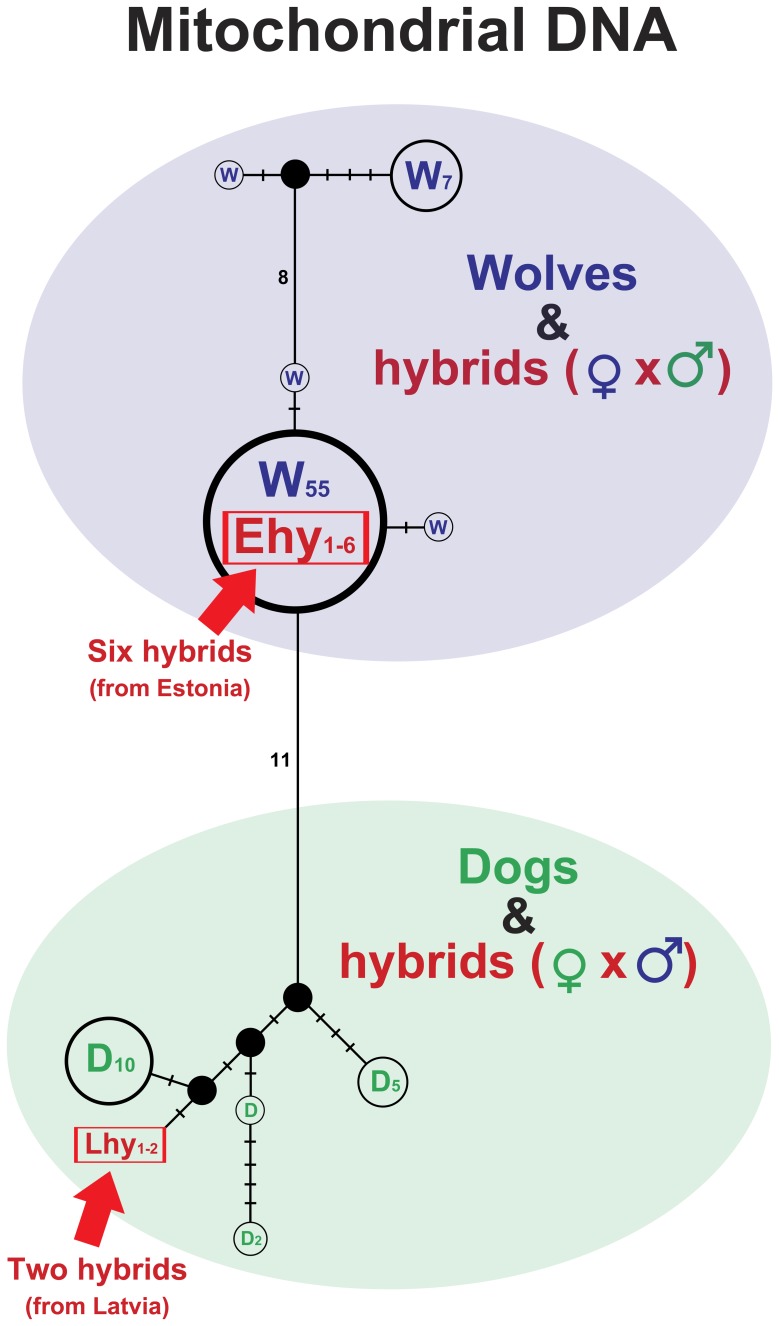
Median joining network of maternal lineages, based on mtDNA control region sequences (1134 bp), showing wolves, dogs and wolf-dog hybrids. Colours: green – dogs, blue – wolves, red – hybrids. W – wolf, D – dog, Ehy (1–6) – six hybrids from Estonia, Lhy (1–2) – two hybrids from Latvia. Numbers of individuals are in parentheses (if more than one individual is represented by the haplotype). Filled circles represent median vectors (haplotypes not sampled or extinct). Short bars indicate single mutations; otherwise the number of mutations is presented (note that the number of mutations and the length of bars are not in proportion).

Wolves and dogs were clearly divided into two distinct haplogroups (Fig. 5). All six hybrids from Estonia carried sequences identical to the major wolf haplotype (n = 55), while the two hybrids from Latvia shared a unique haplotype and grouped together with dogs.

**Figure 6:**
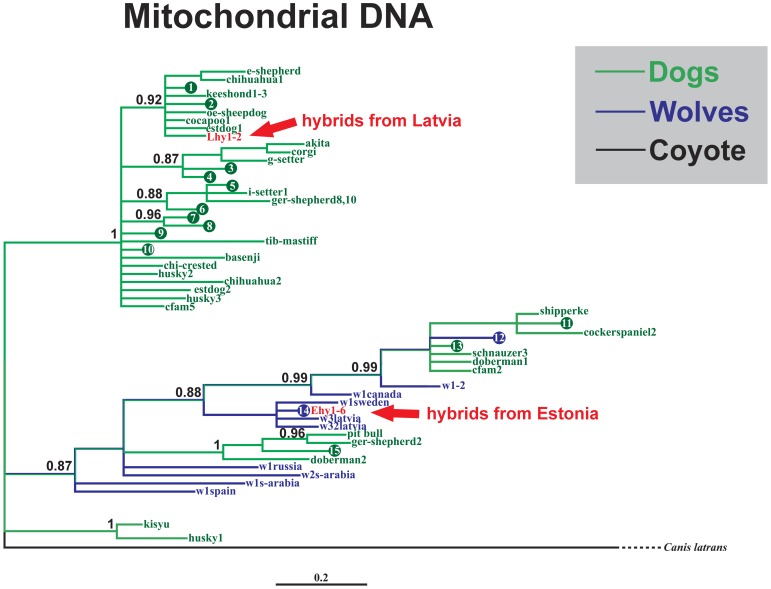
Bayesian phylogenetic tree of wolves, dogs and wolf-dog hybrids (from this study and homologous sequences from GenBank) based on the analysis of mtDNA control region sequences (1134 bp). Nodes with posterior probability values ≥0.80 are shown. Colours: green – dogs, blue – wolves, red – hybrids. Haplotypes representing more than one individual are numbered (circles 1–15). More detailed information about different haplotypes can be found in Table S3.

The larger phylogeny (Fig. 6) which included homologous wolf and dog sequences from GenBank (Table S3) revealed two large clades: one specific to dogs and another that included both wolves and dogs. Both hybrids from Latvia were positioned in the dog-specific clade, while the six hybrids from Estonia were positioned in the wolf-dog clade.

### Paternal network based on variation at seven Y chromosome microsatellite loci

**Figure 7:**
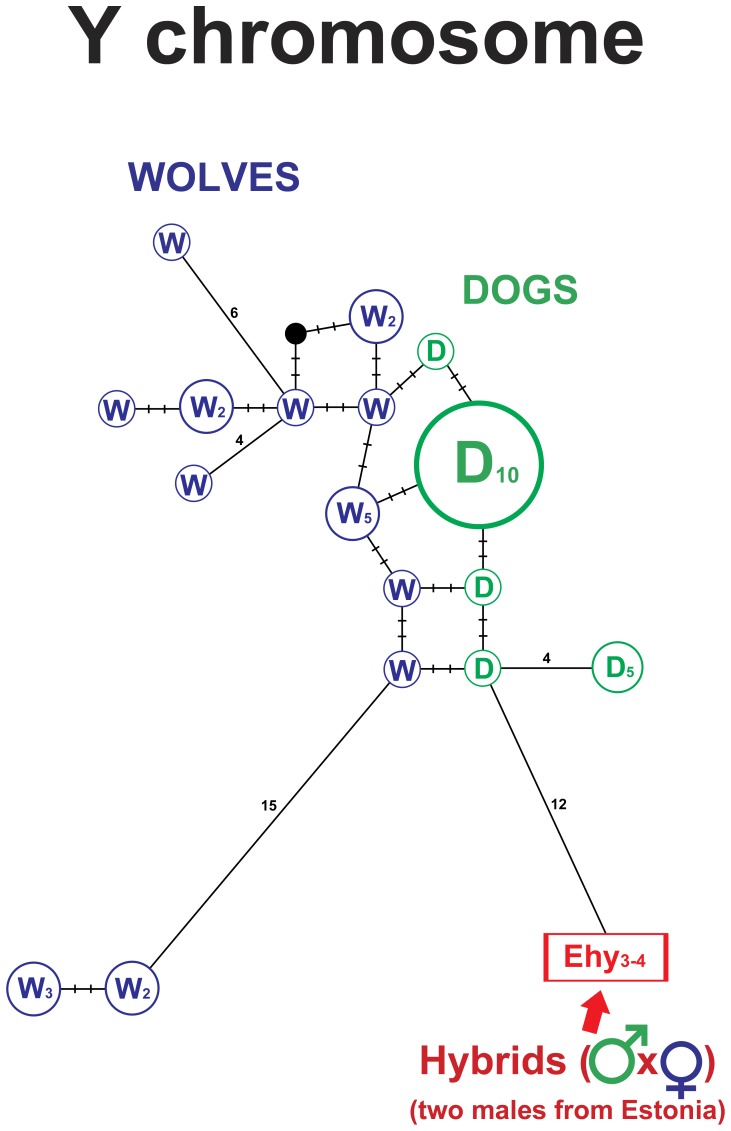
Median joining network of paternal lineages, based on analysis of seven Y chromosome microsatellite loci, showing male wolves, dogs and wolf-dog hybrids. Colours: green – dogs, blue – wolves, red – hybrids. W – wolf, D – dog, Ehy – hybrids from Estonia.

In the whole sample set only two hybrids were males (both from Estonia). Network analysis demonstrated that they were more closely related to dog than wolf haplotypes, suggesting that their paternal lineage was most likely of dog origin (Fig. 7).

## Discussion

### Wolf-dog hybrids in Estonian–Latvian wolf population

While this is the first time that hybridization between gray wolves and dogs has been confirmed in Estonia, it is the second time in Latvia: wolf-dog hybridization was previously reported in Latvia in 1999 and subsequently verified using genetic analysis [16]. The appearance of wolf-dog hybrids in both countries can most likely be explained by the combined effect of two factors: severe and continuous hunting pressure on wolf populations, together with the abundance of stray dogs.

Intensive hunting may have the dual effects of reducing wolf population density and destroying the structure of wolf packs [64], [65]. Some wolves may encounter difficulty of finding a conspecific mate due to the low wolf population density - the Allee effect [66]. If one animal from the alpha pair is removed before mating occurs (the peak of wolf hunting coincides with the wolf mating season in both countries), the remaining animal has to seek another partner in order to reproduce. Where wolf density is low and stray dogs are present, the probability that such a scenario results in hybridization with stray dogs may be increased.

Stray dogs have long been present in Estonia and Latvia, reflecting the common practice of dog-owners in rural areas to let their dogs roam freely. Stray dogs are hunted in both countries, and during 2000–2003 about 1000–1100 dogs were shot annually in Latvia (Latvian official hunting statistics, see also [18]). Since 2004, the number of hunted dogs in Latvia has decreased, staying at approximately 200–250 in 2007–2009. Moreover, the breeding dens of feral dogs have been reported by Latvian State Forest Service rangers from forest in the vicinity of Cesis in the North and Rezekne in the South-East of the country during the last decade (J. Ročāns and J. Mikjanskis, pers. comm.). Although there are no official statistics available for stray dogs in Estonia, they have been abundant for decades (the authors' unpublished observations).

Despite high hunting pressure, the presence of stray dogs and hybridization, this study shows that wolf population has largely remained genetically distinct from dogs in both countries, suggesting that introgressive hybridization in nature might be counteracted by selection or behavioural factors.

### Hybridization between wolves and dogs based on autosomal microsatellite data

Microsatellites have been particularly useful for detecting genetic admixture between wolves and dogs [16]–[21], [27] and in this study hybridization was ascertained through analysis of 11 microsatellite loci. Wolf-dog hybrids exhibited several alleles that were shared with one of the putative parent species but not the other (Fig. S1). The mixed origin of hybrids was also indicated by factorial correspondence analysis, which placed both Estonian and Latvian hybrid clusters as intermediate between wolf and dog clusters (Fig. 2) - a position that would be expected on the basis of their mixed ancestry. Further support for hybrid status was provided by Structure: analysis of the full dataset with ‘parameter set A’ assigned hybrid animals from Latvia with similar membership coefficients to both wolf and dog clusters, while Estonian hybrids received higher assignment values for wolf cluster (Table 2, Fig. 3). This result suggests that hybrids from Latvia are most likely F1, whereas hybrids from Estonia might not be F1 hybrids, but rather F2. On the other hand, analysis with parameter set B assigned all eight Estonian-Latvian hybrids with similar membership coefficients to wolf and dog clusters to (Table 2), indicating that they might be actually F1 hybrids. In addition to Structure, program Newhybrids was also used to assign Latvian and Estonian hybrids, but performed poorly. Moreover, program Newhybrids was equally unable to correctly assign Hybridlab-simulated F1 and F2 hybrids into the proper clusters, whereas Structure was (data not shown) and therefore we consider the results obtained with Structure to be more reliable than those with Newhybrids.

The Structure analysis indicated that ten wolves (three from Estonia and seven from Latvia) exhibited lower membership coefficients of belonging to the wolf cluster than other wolves in this study. Additional attempts to assign these ten animals with Structure and Newhybrids gave inconclusive results, not allowing to make a firm verdict about their filial status and consequently of possible introgression (data not shown). Therefore, analysis with higher number of loci and animals is needed in future studies to investigate introgression.

### mtDNA control region sequences and Y chromosome microsatellite data

While autosomal microsatellite data allowed the existence of wolf-dog hybrids in Estonia and Latvia to be established, gender specific genetic markers were used to evaluate the direction of hybridization. Based on mitochondrial control region data representing the maternal lineage, all six hybrids from Estonia were placed into the wolf haplogroup, carrying sequences identical to a major wolf haplotype (Fig. 5). This suggests that for hybrids collected in Estonia, hybridization took place according to the common pattern, *i.e.* between female wolf and male dog. On the other hand, mtDNA haplotypes found in two Latvian hybrids grouped with dogs, representing an extremely rare case of hybridization between a female dog and a male wolf. This result provides a rare example that violates the general rule of sexual asymmetry in mating between wolves and dogs and it is the first confirmed case from Europe to demonstrate that hybridization has occurred between female dog and male wolf. Although it has been shown that dog haplotypes occasionally fall into the same haplogroup as wolves [e.g. 13], [15], the power to distinguish wolf and dog haplotypes in these cases has been limited by the very short length of mtDNA control region sequences used. Here our results, based on longer mtDNA control region sequences (1134 bp), demonstrate that wolves and dogs form reciprocally monophyletic haplogroups at a local geographic scale, *i.e.* when only wolves and dogs from Latvia and Estonia were represented, and that mtDNA haplotypes of Latvian hybrids clearly group together with dogs (Fig. 5), suggesting that their mother was most likely a dog. On the other hand, when homologous wolf and dog sequences from various other regions were added from GenBank, two clades appeared, one specific to dogs, but in the other both wolves and dogs were present (Fig. 6; note that this is not a definitive phylogenetic network for wolves and dogs, but represents only animals for which homologous sequence of 1134 bp from the mtDNA control region was available in GenBank). In this phylogeny, hybrids from Latvia were placed into the dog-specific clade and hybrids from Estonia into a well-supported subclade of wolves. Thus, despite the lack of reciprocal monophyly, this result also suggests that the mother of Latvian hybrids was a dog, whereas for Estonian hybrids the mother was wolf.

According to the network analysis based on seven Y chromosome microsatellite loci, both male hybrids from Estonia were more related to the dog group (Fig. 7) than to wolves, suggesting that their paternal lineage was likely of dog origin. This is in agreement with the mtDNA analysis, which demonstrated that their maternal lineage was of wolf origin. However, distinction between dog and wolf specific groups was not definitive (they were not monophyletic), and analysis with additional samples and polymorphic Y chromosome microsatellite loci will be required to enhance the resolution of this assay in future investigations.

### Sexual asymmetry in mating between wolves and dogs

To date, field observations have only reported hybridization involving female wolves and male dogs [22]–[25]. The same breeding pattern has also been found in most genetic studies [15], [16], [18]–[21], [26] suggesting that hybridization between gray wolves and dogs is asymmetric. However, as this study (hybrids from Latvia) and a recent report from Canada [27] have shown, this asymmetry is sometimes violated. Munoz-Fuentes *et al.* [27] reported a dog mtDNA haplotype in three individuals from the historic wolf population in Vancouver Island that were morphologically identified as wolves. As the majority of microsatellite alleles were of wolf origin and the minority were shared with dogs, the most likely scenario to explain those data is that hybridization and subsequent introgression had taken place. It has also been shown that the melanistic locus mutation in North American wolves derives from past hybridization with domestic dogs [67]. In Europe, introgressive hybridization has been suggested by a study of the Italian wolf population [18] and in a very recent study from the Iberian Peninsula [20]. As determining introgression is of critical importance, this aspect clearly requires further investigation also in wolf population in Estonian and Latvia.

**Figure 8:**
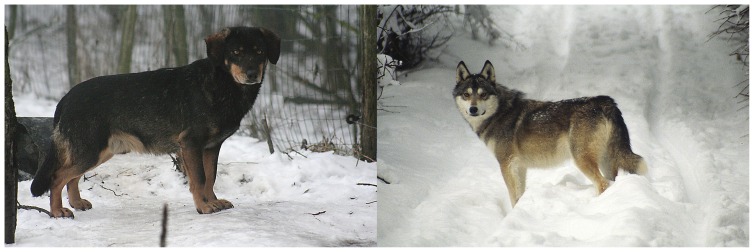
First-generation (F1) wolf-dog hybrids from Wildlife Park Kadzidlowo, Poland: female wolf×male Polish Spaniel (left); female wolf×West Siberian Laika (right) (photos: A. Krzywinski).

The reasons for sexual asymmetry in hybridization between gray wolves and dogs are not clear, though some explanations can be found in literature [13], [27]. There are a number of potentially important factors that may explain why sexual asymmetry has been observed in the direction of hybridization between female wolves and male dogs: 1) adult male wolves frequently prey on dogs [16], [68]–[71] and as a result dogs may avoid contact with them. However, avoidance of female wolves by dogs may not be so pronounced because female wolves are smaller and perhaps less aggressive than males; 2) female wolves also seem to be more active than males in seeking for a dog as a partner. Female wolves have been observed calling for male dogs around villages during the mating period and on one occasion a female wolf was even observed successfully attracting the same male dog in two consecutive years [23]. It has also been noted that female wolves mating with dogs are sometimes injured or old [23], which may reduce their ability to find and/or be accepted by a wolf partner; 3) male dogs are usually capable of mating all year round, which makes them readily available for a female wolf that did not find a wolf partner [see also 13]; 4) hybrid offspring born to a female wolf probably have a greater chance of survival in the wild than those born to a female stray dog. Dogs are almost certainly not as well adapted as wolves for survival in the wild, supported by observations of high pup mortality in stray dogs in Italy [72]; 5) hybrids born to female dogs may be easily overlooked (see examples on Fig. 8, particularly the F1 hybrid of female wolf and male Polish Spaniel) and considered as crosses between different dog breeds if they live around humans or as part of a stray dog pack. Moreover, if female dogs involved in hybridization are not truly feral but just freely ranging, they usually do not bring up their offspring in the wild and hence the hybrid offspring may remain undetected in genetic investigations.

Although many factors seem to favour mating between female wolf and male dog, it is clearly violated sometimes. The simplest imaginable route by which dog mtDNA could be transmitted into the wolf population is direct mating between a male wolf and a female dog. Although such events are probably extremely rare, they may occur when the number of female wolves is low and/or stray dogs are abundant. This scenario is also the most likely explanation for the results of this study for Latvian hybrids (since they carried dog mtDNA and were F1 hybrids according to microsatellite analysis).

### Conservation and management implications

In order to minimize hybridization between wolves and dogs, the most effective strategy would appear to be long-term maintenance of wolf populations at stable densities and with the natural social structure preserved. This is clearly easiest to achieve in large protected populations. However, in hunted populations, in areas with low wolf population density, including the edges of otherwise large and healthy populations, and in the presence of stray dogs (such as in Estonia and Latvia) wolf hunting should be prohibited and the population closely monitored with respect to hybridization and introgression using noninvasive sampling. Moreover, in such areas local people should be instructed, using also legislative tools if necessary, to keep their dogs from ranging freely, especially during the wolf mating period.

### Conclusions

In this work, hybridization between gray wolf and domestic dog was ascertained in Estonia (for the first time) and Latvia (second time) using a combined analysis of maternal, paternal and biparental genetic markers. Six hybrid individuals from Estonia and two from Latvia were initially detected from their atypical morphological traits and their hybrid status was subsequently confirmed using genetic analysis. Analysis of mtDNA showed that the two hybrids from Latvia represented a very rare case of hybridization – the first record from Europe – between a female dog and a male wolf. Latvian hybrids were determined as F1 and and the filial generation status of hybrids from Estonia was probably also F1, though this result was equivocal.

Despite hybridization, the genetic integrity of wolf populations in Estonia and Latvia does not seem to be severely threatened at the moment. However, due to the danger posed by stray dogs, hunting pressure on wolves should be kept under control, especially in areas with low wolf abundance, to keep the hybridization rate as low as possible.

## Supporting Information

Figure S1 **Distribution of allele frequencies at 11 autosomal microsatellite loci in 74 wolves from Estonia and Latvia (blue bars), 21 pure-bred dogs (red bars) and eight wolf-dog hybrids (green bars) from Estonia and Latvia.** The horizontal scales indicate base pair lengths of the different alleles; vertical scales indicate the relative allele frequencies. The most pronounced alleles exhibited by hybrids and also found in wolves but absent in dogs, and *vice versa*, are circled.
(PDF)

Table S1 **The 11 autosomal microsatellite (in bold) and seven Y chromosome microsatellite loci used.**
(PDF)

Table S2 **Kinship analysis: sibling relationships among Estonian and Latvian hybrids using the the relatedness estimator according to Konovalov and Heg.** Calculations were conducted using Kingroup v2.0. Full-siblings are indicated in bold.
(PDF)

Table S3 **Wolves, dogs and wolf-dog hybrids used for Bayesian phylogenetic analysis (based on partial mtDNA control region sequences, 1134 bp;** **Fig. 5** **).**
(XLS)
