# Supplementary material for: Bucking the Trend in Wolf-Dog Hybridization: First Evidence from Europe of Hybridization between Female Dogs and Male Wolves
Source: PLoS One. 2012 Oct 3;7(10):e46465. doi: 10.1371/journal.pone.0046465 (PMC3463576; doi:10.1371/journal.pone.0046465)
Supplement: Figure S1 — Distribution of allele frequencies at 11 autosomal microsatellite loci in 74 wolves from Estonia and Latvia (blue bars), 21 pure-bred dogs (red bars) and eight wolf-dog hybrids (green bars) from Estonia and Latvia. The horizontal scales indicate base pair lengths of the different alleles; vertical scales indicate the relative allele frequencies. The most pronounced alleles exhibited by hybrids and also found in wolves but absent in dogs, and vice versa, are circled. (PDF) [file pone.0046465.s001.pdf]

Fig. S1. Distribution of allele frequencies at 11 autosomal microsatellite loci in 74 grey wolves from the Estonian-Latvian wolf population (blue bars), 21 pure-bred dogs (red bars) and eight wolf-dog hybrids (green bars) from Estonia and Latvia. The horizontal scales indicate the molecular weights in base pair lengths of the different alleles; vertical scales indicate the relative allele frequencies. Most pronounced alleles exhibited by hybrids and also found in wolves but absent in dogs, and *vice versa*, are surrounded.

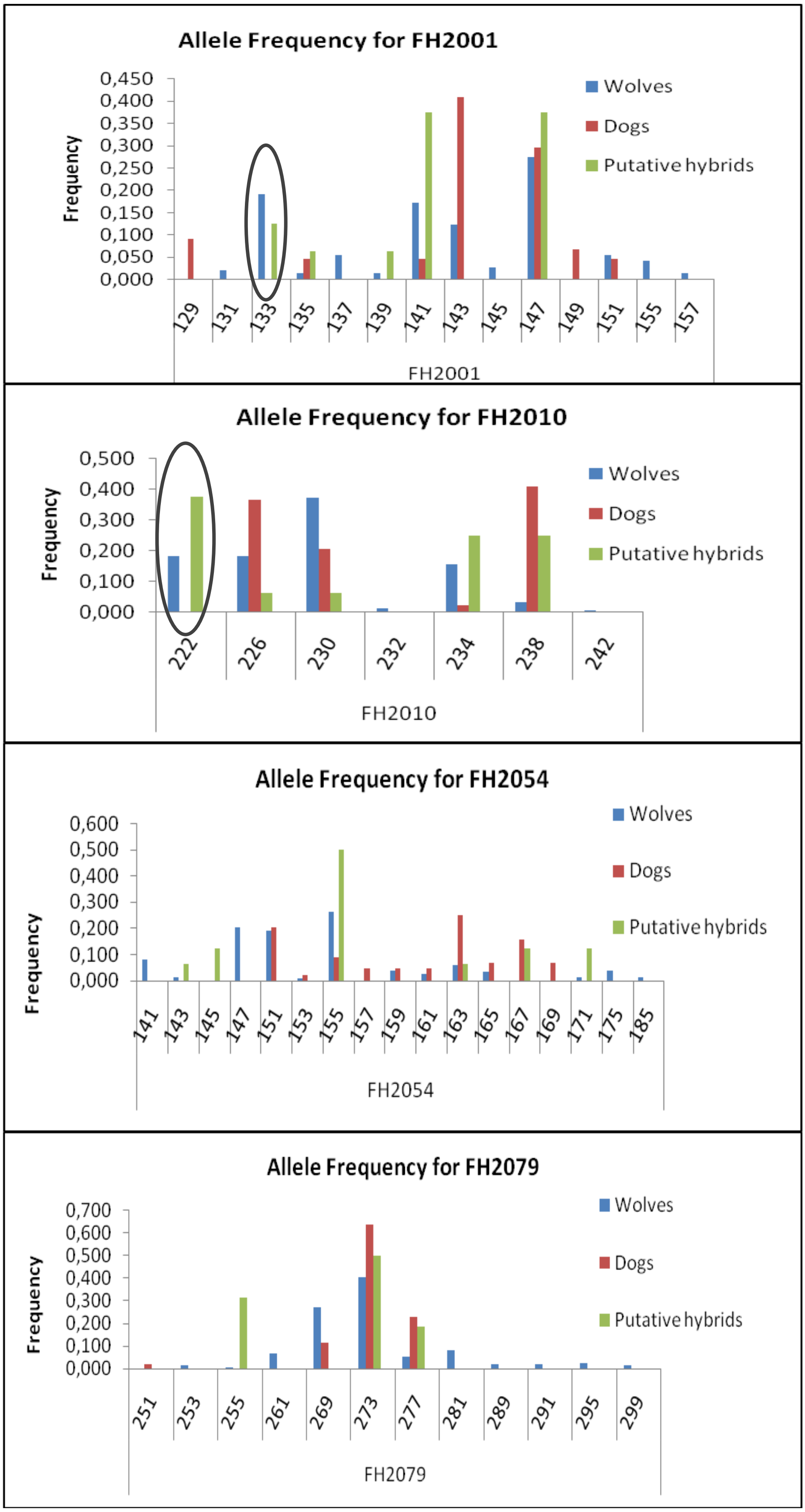

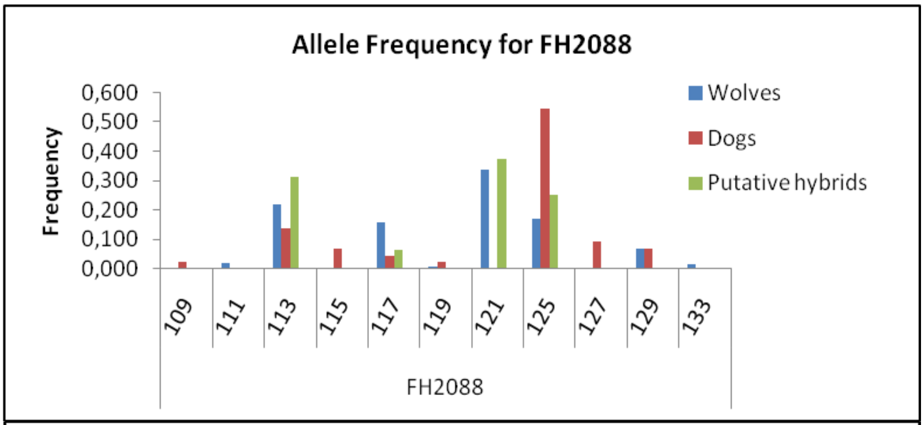
Allele Frequency for FH2088
Frequency
0,600
0,500
0,400
0,300
0,200
0,100
0,000
109
111
113
115
117
119
121
125
127
129
133
FH2088
Wolves
Dogs
Putative hybrids

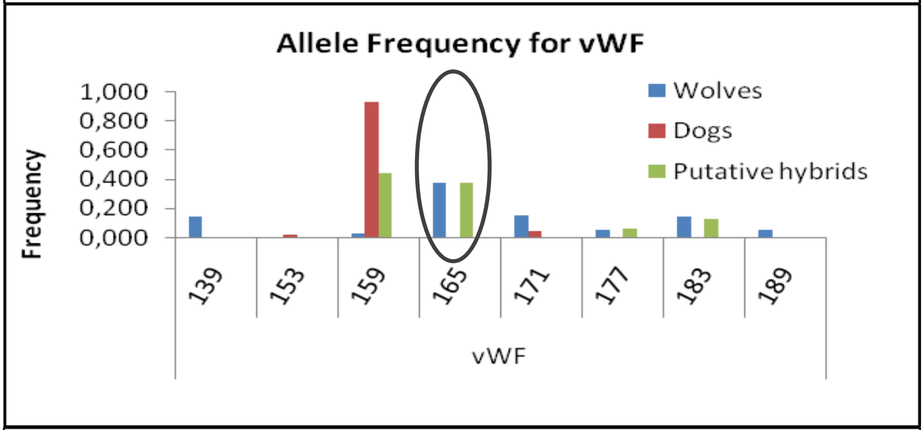
Allele Frequency for vWF
Frequency
1,000
0,800
0,600
0,400
0,200
0,000
139
153
159
165
171
177
183
189
vWF
Wolves
Dogs
Putative hybrids

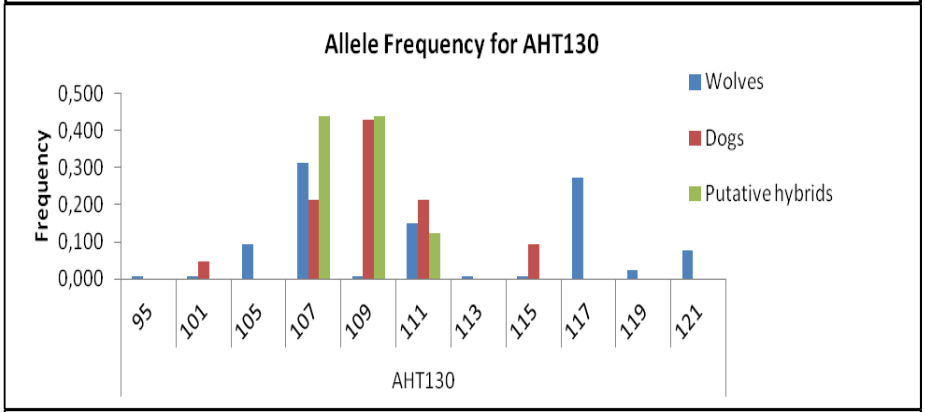
Allele Frequency for AHT130
Frequency
0,500
0,400
0,300
0,200
0,100
0,000
95
101
105
107
109
111
113
115
117
119
121
AHT130
Wolves
Dogs
Putative hybrids

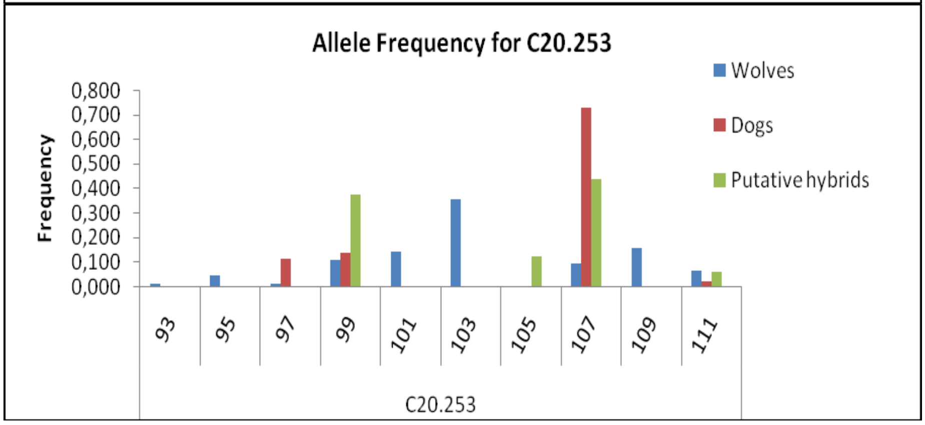
Allele Frequency for C20.253
Frequency
0,800
0,700
0,600
0,500
0,400
0,300
0,200
0,100
0,000
93
95
97
99
101
103
105
107
109
111
C20.253
Wolves
Dogs
Putative hybrids

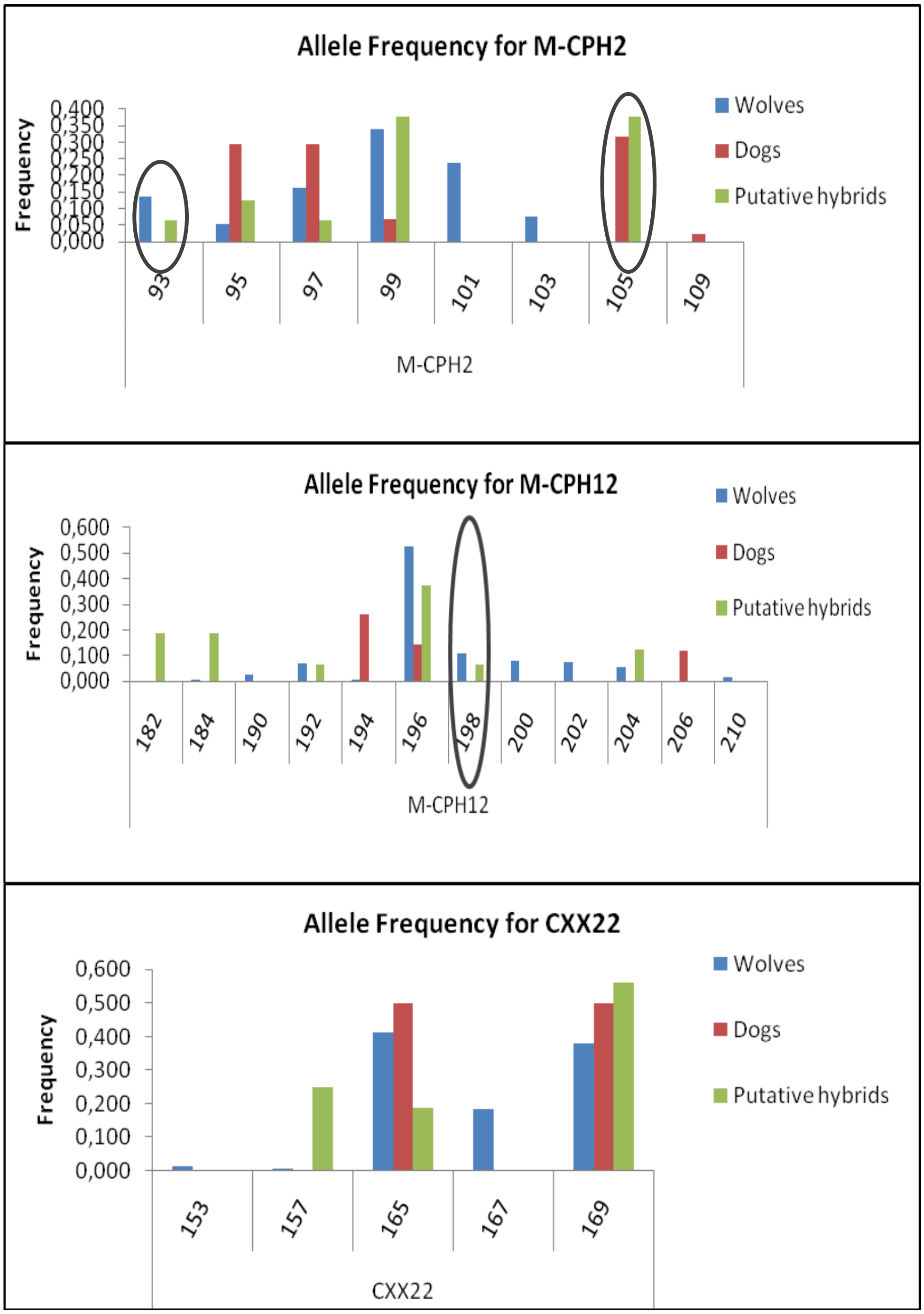
Allele Frequency for M-CPH2
Frequency
0,400
0,350
0,300
0,250
0,200
0,150
0,100
0,050
0,000
93
95
97
99
101
103
105
109
M-CPH2
Wolves
Dogs
Putative hybrids
Allele Frequency for M-CPH12
Frequency
0,600
0,500
0,400
0,300
0,200
0,100
0,000
182
184
190
192
194
196
198
200
202
204
206
210
M-CPH12
Wolves
Dogs
Putative hybrids
Allele Frequency for CXX22
Frequency
0,600
0,500
0,400
0,300
0,200
0,100
0,000
153
157
165
167
169
CXX22
Wolves
Dogs
Putative hybrids
