# Supplementary material for: Bucking the Trend in Wolf-Dog Hybridization: First Evidence from Europe of Hybridization between Female Dogs and Male Wolves
Source: PLoS One. 2012 Oct 3;7(10):e46465. doi: 10.1371/journal.pone.0046465 (PMC3463576; doi:10.1371/journal.pone.0046465)
Supplement: Table S1 — The 11 autosomal microsatellite (in bold) and seven Y chromosome microsatellite loci used. (PDF) [file pone.0046465.s002.pdf]

Table S1. The 11 autosomal microsatellite (in bold) and seven Y chromosome microsatellite loci used

Locus	Primer pair	Length of alleles (bp)	Dye (forward primer)	Reference
**FH2001**	For - TCCTCCTCTTCTTTCCATTGG<br>Rev - TGAACAGAGTTAAGGATAGACACG	129-149	6-FAM	[1]
**FH2010**	For - AAATGGAACAGTTGAGCATGC<br>Rev - CCCCTTACAGCTTCATTTTCC	203-235	PET	[1]
**FH2054**	For - GCCTTATTCATTGCAGTTAGGG<br>Rev - ATGCTGAGTTTTGAACTTTCCC	146-178	6-FAM	[1]
**FH2079**	For - CAGCCGAGCACATGGTTT<br>Rev - ATTGATTCTGATATGCCCAGC	261-285	6-FAM	[1]
**FH2088**	For - CCTCTGCCTACATCTCTGC<br>Rev - TAGGGCATGCATATAACCAGC	104-136	VIC	[1]
**vWF**	For - CTCCCCTTCTCTACCTCCACCTCTAA<br>Rev - CAGAGGTCAGCAAGGGTACTATTGTG	129-189	PET	[2]
**AHT130**	For - CCTCTCCTGGTAATTGCTGC<br>Rev - TGGAACACTGGTCCCCAG	108-124	NED	[3]
**C20.253**	For - AATGGCAGGATTTTCTTTTGC<br>Rev - ATCTTTGGACGAATGGATAAGG	93-115	6-FAM	[4]
**CXX22**	For - AGCGACTATTATATGCCAGCG<br>Rev - CTCATTGGTGTAAAGTGGCG	163-169	PET	[4]
**M-CPH2**	For - TTCTGTTGTTATCGGCACCA<br>Rev - TTCTTGAGAACAGTGTCCTTCG	95-109	VIC	[5]
**M-CPH12**	For - TTCTGTTGTTATCGGCACCA<br>Rev - GATGATTCCTATGCTTCTTTGAG	193-215	VIC	[5]
MS34A	For - AGCCATTCCTGGCCGAGTGG<br>Rev - GGTCCCCTTTTGGCATAGTGT	172–178	6-FAM	[6]
MS34B	For - AGCCATTCCTGGCCGAGTCC<br>Rev - GGTCCCCTTTTGGCATAGTGT	174-182	PET	[6]
990-35	For - CCATCCGCAGAACAGGTATT<br>Rev - GGGCCGCTATTTTAGGTGAT	125-131	6-FAM	[6]
MS41A	For - TCCTCTAATTTTCCCCTCTA<br>Rev - CTGCTCGACCCTCTTCTCTG	208-210	VIC	[6]
MS41B	For - TCCTCTAATTTTCCCCTCTC<br>Rev - CTGCTCGACCCTCTTCTCTG	212-230	6-FAM	[6]
650-792	For - ACTCAGCTCTCCCTTGTCCA<br>Rev - TTGAGGGCAGGTCTCAGAGT	128-136	PET	[7]
650-793	For - AGTTTCTGCCCAGGAAGGAC<br>Rev - AGCTGAGCGGTTTGAAACTT	124-134	VIC	[7]

References

1. Francisco LV, Langston AA, Mellersh CS, Neal CL, Ostrander EA (1996) A class of highly polymorphic tetranucleotide repeats for canine genetic mapping. Mamm Genome 7: 359–362.
2. Shibuya H, Collins BK, Huang THM, Johnson GS (1994) A polymorphic (AGGAAT)n tandem repeat in an intron of the canine von Willebrand factor gene. Anim Genet 25: 122.
3. Holmes NG, Dickens HF, Parker HL, Binns MM, Mellersh CS *et al*. (1995) Eighteen canine microsatellites. Anim Genet 25: 132-133.
4. Ostrander EA, Sprague GF, Rine J (1993) Identification and characterization of dinucleotide repeat (CA)n markers for genetic mapping in dog. Genomics 16: 207–213.
5. Fredholm M, Winteroe AK (1995) Variation of short tandem repeats within and between species belonging to the Canidae family. Mamm Genome 6: 11–18.
6. Sundqvist A-K, Ellegren H, Olivier M, Vilà C (2001) Y chromosome haplotyping in Scandinavian wolves (*Canis lupus*) based on microsatellite markers. Mol Ecol 10: 1959–1966.
7. Bannasch DL, Bannasch MJ, Ryun JR, Famula TR, Pedersen NC (2005) Y chromosome haplotype analysis in purebred dogs. Mamm Genome 16: 273–280.
