# Supplementary material for: Bucking the Trend in Wolf-Dog Hybridization: First Evidence from Europe of Hybridization between Female Dogs and Male Wolves
Source: PLoS One. 2012 Oct 3;7(10):e46465. doi: 10.1371/journal.pone.0046465 (PMC3463576; doi:10.1371/journal.pone.0046465)
Supplement: Table S2 — Kinship analysis: sibling relationships among Estonian and Latvian hybrids using the the relatedness estimator according to Konovalov and Heg. Calculations were conducted using Kingroup v2.0. Full-siblings are indicated in bold. (PDF) [file pone.0046465.s003.pdf]

**Table S2.** Kinship analysis: sibling relationships among Estonian and Latvian hybrids using the the relatedness estimator according to Konovalov and Heg. Calculations were conducted using Kingroup v2.0. Full-siblings are indicated in bold.

| Individual 1 | Individual 2 | P-values |
|---|---|---|
| Ehy2 | Ehy1 | **0,50** |
| Ehy3 | Ehy2 | **0,50** |
| Ehy4 | Ehy2 | **0,50** |
| Ehy5 | Ehy2 | **0,50** |
| Ehy6 | Ehy1 | **0,50** |
| Ehy6 | Ehy3 | **0,50** |
| Ehy6 | Ehy4 | **0,50** |
| Ehy6 | Ehy5 | **0,50** |
| Ehy3 | Ehy1 | 0,063 |
| Ehy4 | Ehy1 | 0,063 |
| Ehy4 | Ehy3 | 0,063 |
| Ehy5 | Ehy1 | 0,063 |
| Ehy5 | Ehy3 | 0,063 |
| Ehy5 | Ehy4 | 0,063 |
| Ehy6 | Ehy4 | 0,063 |
| Lhy1 | Lhy2 | **0,50** |
